# A Reference Hand-Book of the Medical Sciences

**Published:** 1904-10

**Authors:** 


A Reference Hand-Book of the Medical Sciences.—Embracing the entire range of scientific and practical medicine and allied science. By various writers. A new edition, completely revised and rewritten. Edited by Albert H. Buck, M. D., New York City. Illustrated by chromolithographs and six hundred and eighty-eight half-tone and wood engravings. New York: Wm. Wood & Co. 1904.

This is volume VII, and in every way it equals its predecessors. The contents run from saccharin to ulcer and covers many of the best subjects in medicine. As has been said before, in this department of the other volumes is no work extant which covers a broader scientific field and meets the demands for a reliable reference as well as the reference hand-book. It is indexed systematically, arranged practically and illustrated in hundreds of ways to satisfy the professional man. The very subject the doctor has looked over his library for and has failed to find any reference to is found in the reference hand-book. For example, look for the tongue, all it shows in disease; look for suicide, a scientific and statistical article; look for health resorts in the world, or any other out-of-the-way subject, and the reference hand-book never disappoints you.

T. J. B.

Notice of the last volume (VIII) of the series appeared in September number.

